# Using an interactive digital calendar with mobile phone reminders by senior people - a focus group study

**DOI:** 10.1186/s12877-019-1128-9

**Published:** 2019-04-23

**Authors:** Vedrana Baric, Maria Andreassen, Annika Öhman, Helena Hemmingsson

**Affiliations:** 1 Department of Social and Welfare Studies, Division of Occupational Therapy, Linköping University, Norrköping, Sweden; 2 Department of Special Education, Stockholm University, Stockholm, Sweden

**Keywords:** Technology, Active reminders, Mobile/smartphone, Self-help devices, Reminder systems, Cell phone, Text messaging, Habits

## Abstract

**Background:**

New technologies such as mobile/smartphones have the potential to help senior people perform everyday activities. However, senior people may find it difficult using mobile/smartphones, especially the digital calendar and short text message features. Therefore, senior people might need user-friendly, flexible, and interactive digital calendars that provide them with active reminders about their everyday activities. This study focuses on community dwelling seniors’ experiences learning and using RemindMe, an interactive digital calendar with active reminders, as part of customizing an intervention appropriate for senior people with cognitive impairments.

**Methods:**

Four focus groups were conducted with 20 community dwelling seniors (11 men and 9 women) who all had used RemindMe for six weeks. The focus groups were tape recorded, transcribed verbatim, and analysed using content analysis.

**Results:**

For participants in this study, using a calendar was an essential part of their everyday lives, but only a few had experiences using a digital calendar. Although the participants described RemindMe as easy to use, they had a difficult time incorporating RemindMe into their daily routines. In part, these difficulties were the result of the participants needing to change their mobile/smartphone routines. Some participants felt that using an interactive digital calendar was a sign of modernity allowing them to take part in the society at large, but others felt that their inability to use the technology was due to their age, dependence, and loss of function. Participants found that receiving active reminders through short text messages followed by actively acknowledging the reminder helped them perform more everyday life activities. This feature gave them a higher sense of independence and control.

**Conclusions:**

Community dwelling seniors found that RemindMe was easy to learn and to use, although they also found it challenging to integrate into their everyday lives. For senior people to make the effort to develop new routines for mobile/smartphone use, a prerequisite for using a digital calendar, they need to be motivated and believe that the technology will make their lives better.

## Introduction

Over the past decades, mobile phones and smartphones have expanded the way people perform everyday life tasks as well as seek, acquire, and communicate information [1]. While much is known about how young people are accustomed to using mobile phones and smartphones, less is known about how senior people use mobile phones and smartphones. This knowledge gap needs to be closed as new technologies such as tablets, the Internet, and mobile/smartphones may be used to support seniors live independently in their own homes by providing several practical benefits such as alarms, reminders, and pre-programmed mobile numbers [2]. For example, Jamison et al. [3] found that people with memory impairment benefit from using prompting technology. These technologies can provide cognitive support and enhance the possibility to be independent in everyday life for people with cognitive impairments as the result of stroke, traumatic brain injury, or other diagnoses that may affect memory or executive functioning (i.e., the ability to plan and structure everyday life) [4–8].

Technology with active reminders that provide prompts/reminders of scheduled activities/events can enhance people’s ability to perform activities in everyday life [9]. However, passive reminders (e.g., calendars, diary, Post-it, and notebooks) require the user to remember look in a calendar or notebook for information [10]. Thus, using digital reminders in mobile/smartphones can provide cognitive support and may improve performance and task completion rates in everyday life for people with acquired brain injuries, dementia, or cognitive difficulties [4, 5, 10, 11]. In addition, senior people at risk for cognitive impairments may benefit from having access to digital technology as it can enable everyday life activities and support independence [12, 13].

In general, seniors are embracing new technologies, emphasizing the benefits of enhanced safety and social interactions [13, 14], and seem to have a positive attitude toward technology, a finding that contradicts the prevalent idea that senior people are negative toward new technology [13, 15]. However, seniors are also likely to face challenges when getting older that include lack of familiarity with and access to technology, discomfort asking for support, and issues of trust, privacy, and ease of use [16, 17]. Senior people may also experience other barriers for technology use. Senior people may find seeing and handling a device difficult due to visual or physical impairments. In addition, hearing impairments may make it difficult for some seniors to hear alarms or signals [18]. Until recently, available technology that prompts or reminds users of activities/events has been specifically developed as assistive devices, requiring the user to learn to use the technology and most often provide time-based prompts/reminders without delivering reminders that take the user’s environment into consideration [9, 16]. Therefore, it is recommended that prompts/reminders should be incorporated into mobile/smartphones to provide more precise location-related reminders [9]. Additionally, prompt/reminders need to be flexible and have the capability to deal with changing situations in time or place, due to the user’s everyday life. Providing training and support could make devices easier to use as well as improve the way people are trained to use the technology [16, 17].

Several memory aids and prompting/reminder systems have been developed to support people with cognitive impairments perform daily activities in everyday life such as personal digital assistant (PDA) [7], pagers, [11] web-based calendars such as Google calendar [10] and other prompting technologies [4, 9, 19] as well as mobile phone applications [20, 21]. However, pagers, web-based calendars, and PDAs using “active reminders” to alert users to complete activities at specified times [7] have some drawbacks. For example, many of these are linked to specific personal computers or specific mobile/smartphone brands that might require a long learning and implementation time for users as well as provide only limited opportunities for feedback on activities performed [7]. To overcome some of the limitations of existing memory aids and prompting systems, RemindMe, an interactive digital calendar with mobile phone reminders, was recently developed and designed to support people in organizing, planning, and executing activities in everyday life. RemindMe offers three core functions: (i) scheduling of activities/events, and reminders with a user-friendly web-based calendar; (ii) active conformation of reminders sent by short text messages (SMS); and (iii) a unique feedback system that registers self-monitoring information based on the user’s interaction with the system. The core functions of RemindMe offer several additional advantages including using active confirmation of reminders requiring users to actively acknowledge the prompt by responding to a SMS. Active confirmation of reminders has been described as particularly useful for supporting performance of activities in everyday life for people with cognitive impairments [22] by increasing awareness of activities/events needed to be performed [21]. RemindMe also provides users with a unique feedback system that registers self-monitoring information based on the user’s interaction with the system.

The first step in the development of RemindMe included identifying technical errors and barriers to user interface in order to optimize the system before further evaluation. This was performed from the perspective of adult users who used RemindMe for four weeks and reported all identified technical errors and barriers to user interface that occurred when using RemindMe. In addition, to Remind Me’s technical stability consideration has been given to the usability and satisfaction with RemindMe, since this is a strong predictor of the user’s acceptance or rejection of a device [23]. Hence, in the second step of the developmental process, RemindMe’s usability was examined from the perspective of professionals supporting people with cognitive disabilities perform everyday life activities [21]. By investigating the usability of RemindMe from the perspective of professionals, initial understanding of the general needs of reminder systems used in rehabilitation with individuals with cognitive impairments was obtained [21]. The current study further explores the everyday use of RemindMe from the perspective of community dwelling seniors to understand how senior people experience learning and using RemindMe before RemindMe is further evaluated as part of customizing an intervention appropriate for senior people with cognitive impairment.

### Aim

This study explores senior peoples’ experiences learning and using RemindMe, an interactive digital calendar with mobile phone reminders.

## Method

### Study design

The study uses focus group interviews [24] involving senior people who had volunteered to learn and use RemindMe for six weeks.

### The interactive digital calendar with SMS reminders (RemindMe)

RemindMe was developed to offer easy and interactive scheduling of activities/events and reminders by using a user-friendly digital calendar designed to reduce complexity, including limiting choice of colours and limiting options for multiple windows [1]. RemindMe uses active reminders sent by a SMS that can be set by a support person such as family members or a professional using a digital calendar. The user invites a support person to share the digital calendar for as long as the user needs the support. The user can customize the reminders in the following ways: when to send the reminder – once, daily, weekly, monthly, and yearly; who receives the reminder; and where the event is located. RemindMe sends a SMS to the users’ own mobile or smartphone with a reminder of the scheduled activity and a request of an active response by answering (Yes/No) to the reminder. The scheduled activity/event as well as the user’s answer are recorded in RemindMe’s feedback system, which keeps a complete personalized history of all activities/events – past, present, and future – based on the user’s interaction with the system. The feedback system offers a quick view of dates and times of all completed activities/events, using no more than three colours to display all completed actions and uncompleted actions, including whether the user answered the SMS reminder. In addition, the feedback system offers information on who, either the person or support person, scheduled the activity/event and the number of weeks RemindMe was used. This study does not report on data from the feedback system.

### Introduction course

To help seniors learn how to use RemindMe, the senior people were provided with an introduction course, approximately 30–90 min, on how to use RemindMe. Two introduction courses were held in computer rooms at a local library or university. Some participants brought their own laptop or iPad. In addition, all participants were provided with a written manual on how to use the core functions of RemindMe. During the following six weeks, the participants also received a weekly support phone call from the same research assistant. These phone conversations targeted their use of RemindMe and potential difficulties. During the introductory course, participants answered a short questionnaire developed for the present study that included demographic questions, current perceived impairments, and frequency of computer and mobile and/or smartphone use.

### Participants

Convenience sampling [23] was used to recruit participants through the Swedish National Pensioners’ Organization (PRO), which is the largest organisation for pensioners in Sweden [25]. The study was presented at regular PRO meetings in four different housing areas in two different cities in south-eastern Sweden. Inclusion criteria for the focus groups were being at least 65 years of age, having access to a mobile phone or smartphone and a computer or tablet, and willing to try RemindMe for six weeks. In addition, the participants agreed to participate in a focus group interview to discuss their experiences using RemindMe in everyday life. At the meeting, 23 seniors who fulfilled all the inclusion criteria registered their interest and were invited and participated in the introduction courses. Three people were unable to participate in the final focus group interviews. Hence, 20 senior people who had used RemindMe for six weeks participated in the focus group interviews and completed the study. The participants’ ages ranged from 66 to 85 (mean age of 73) and there were almost as many women (45%) as men (55%) (Table 1). Most participants (85%) reported mobile phone use on a daily or weekly basis and about half (55%) used a computer on a daily or weekly basis. Several participants reported hearing and/or vison impairments.

**Table 1:** Descriptions of participants: gender, level of education, age, living status, self-reported impairments and current use of technology

		n (%)
Gender	Female	9 (45)
	Male	11 (55)
Level of education	Compulsory school (6–9 years)	7 (35)
	Upper secondary school (12–14 years)	5 (25)
	University, University Colleges, Higher Vocational Education	8 (40)
Age	Mean	73,7 (SD = 5.16; Range 66–85)
Living status*^1^	Single	4 (20)
	Married	11 (55)
	Partner but living apart	3 (15)
Self-reported	Reduced vision	6 (30)
Impairments*^2^	Reduced hearing	10 (50)
	Reduced memory	3 (15)
	None of these impairments	6 (30)
Current use of technology	Daily or weekly computer use	11 (55)
	Daily or weekly mobile phone use	17 (85)

n = number of respondents; *^1^ = missing = 2; ^*2^ = the participants could tick several options

### Data collection

#### Focus group interviews and procedure

In total, 20 senior people participated in four focus group interviews (11 men and 9 women), with five participants in each group (Table 2). The focus groups were organized based on geographical areas and met in places close to the participants’ homes – a university (one group), a library (one group), and meeting rooms in the community arranged by the participants’ themselves (two groups). All participants organized their own transportation.

**Table 2:** Overview of the participants in the four focus groups

Group1		Group 2		Group 3		Group 4	
Female	66	Female	70	Male	71	Male	76
Male	66	Male	85	Male	76	Male	74
Female	79	Male	71	Male	73	Male	81
Female	73	Male	77	Male	82	Female	70
Female	71	Female	75	Female	69	Female	70

The focus groups started with the participants being greeted, offered a snack, and asked to sit at a round table. All focus groups were led by a moderator (the first author) to ensure uniformity between focus groups [26]. In addition, an assistant moderator was responsible for note taking and follow-up questions when needed. The moderator told the participants about the purpose of the study and the voluntary nature of the group. The participants gave their oral consent to participate in the interview. This interaction was tape recorded. All focus group interviews started with an introduction that explained that the emphasis was on the participants’ discussion and conversation with each other [26]. Participants were encouraged to openly present their views and discuss with one another their experiences using RemindMe in everyday life.

The main questions targeted the potential usefulness as well as shortcomings of the core functions of RemindMe. The moderator emphasized that both positive and negative experiences were of interest. After the moderator’s introduction, the participants were asked to introduce themselves to the group. Next, the participants were asked to discuss the following introductory interview questions: (1) What were your experiences using RemindMe (i.e., learning to use new technology, its usefulness, and its barriers)?; (2) What were your experiences using the three core functions of RemindMe (i.e., scheduling of the activities/events and reminders, the active confirmation of SMS, and the feedback system)?; (3) What were your experiences when you received support (i.e., the written manual, the introduction course, and the weekly phone support)? These three questions were asked and discussed at every focus group. Follow-up questions were used by the moderator when needing to inquire about the answers to these questions. Probing questions such as “please tell me more” and “please give me an example” were used in connection to the main introductory questions and follow-up questions. Prompts such as “you mentioned earlier” were mainly used by the assistant moderator to trigger the participants to tell more about earlier expressed places, people, and experiences [26]. All interviews were audio taped and lasted between 56 and 65 min, resulting in 100 pages of text transcribed verbatim using Times New Roman 12 font throughout with 1.0 line spacing.

### Analysis

The focus group interviews were analysed using content analysis [27]. All focus group interviews were transcribed and read several times separately by all authors. Next, the first author (VB), second author (MA), and the last author (HH) identified meaning units, which consisted of text passages with similar meaning. The identified meaning units were abstracted and shortened using a description close to the text, referred to as condensed meaning units. Differences and similarities in the condensed meaning units were discussed with the third author (AÖ). Following the joint discussion, the authors agreed on condensed meaning units and labelled these with codes that were close to the participants’ original descriptions. The codes were then checked against the meaning units several times by the first, second, and third authors. In the next step, the codes were sorted and abstracted into subcategories based on descriptions of similarities and differences as well as challenges and benefits using new technology. Categories emerged by incorporating similar sub-categories under one label. The emerging categories were continuously critically discussed by VB, MA, AÖ, and HH and further revised when similarities and differences emerged that could categorize the data. The final categories were labelled and agreed upon by all the authors.

### Trustworthiness

Credibility was attained by involving all co-authors throughout the analysis in discussions of the findings and in the search for meaning units, subcategories, and categories [28]. The analysis contained a back and forth between the transcripts and emerging codes, subcategories, and categories. Quotations from the transcribed text are used to enhance credibility [29]. Detailed descriptions of the selection and characteristics of the participants in terms of age, living status, level of education, self-reported impairments, and frequency of computer and mobile/smartphone use are presented, with the intention of enabling the reader to assess how transferable the results are to other contexts [23, 29]. Dependability was attained by the first, second, and last authors dealing individually with data and this was followed by continuous discussions. Conformability was attained by using the third author (AÖ) to critically check the agreement between the codes, subcategories, and categories.

### Ethical considerations

The study was designed and carried out in accordance with the ethical guidelines of Swedish Research Council [30] and the ethical standards of the Declaration of Helsinki [31]. When the participants volunteered to participate in the study, they received an information letter explaining the aim of the study, the procedure of inclusion, and how the results would be presented. In addition, the letter informed them that participation was voluntary and that they were able to withdraw from the study at any time without any explanation. Before each focus group interview, the participants were presented with oral information about the study and that participation was voluntary. In addition, they were given the opportunity to ask questions. The participants gave their informed consent verbally before the interview and this consent was tape recorded. All data were treated confidentially. The names of the participants have been altered to ensure confidentiality.

## Results

The participants’ experiences using RemindMe are presented as four different themes (Table 3). The first theme focuses on the participants’ experiences with the use of calendars in everyday life and includes one category. The second theme focuses on the symbolic value participants place on using RemindMe in everyday life and includes two categories. The third theme focuses on the participants’ experiences learning to use RemindMe and incorporating it into their everyday lives and includes two categories. The fourth theme focuses on the specific features of RemindMe and includes two categories.

**Table 3:** Themes and categories describing senior people's experiences using RemindMe in everyday life

Theme	Using calendars to organize time and to remind of events in everyday life	Symbolic value	Easy to learn but challenging to integrate in everyday life	Active reminders in everyday life
Categories	Stationary vs. mobile calendar and mobile phone use	A sign of modernity	Learning to use RemindMe despite age-related difficulties	It talks to me
		A sign of aging, dependence, and memory deficits	RemindMe challenges habits	Not worth it for me

### Using calendars to organize time and to remind of events in everyday life

All participants used some type of calendar to plan and organize time and to schedule and remember coming activities in their everyday life. They used different calendars for different purposes related to habits in everyday life, how active the participants were outside of their home, their cognitive ability, and their need for coordination with other people.

#### Stationary vs. mobile calendar and mobile phone use

To have and use a calendar and mobile phone seemed to be an essential part of everyday life for the participants. The participants described using large stationary calendars and mobile phones in their homes. The main reasons for using a home bound non-digital weekly/monthly calendar (e.g., placed on the refrigerator) were that these calendars provided a quick and easy reminder and overview of the week’s activities. They could further be used by those who lived with a partner to inform and remind each other about different activities they should attend individually or together. In addition, pocket calendars were described as providing room for reflections and notes in addition to scheduling activities; that is, they could also be used as a diary:

*“No, you have to have one like this [a pocket weekly calendar] because you almost always need to write memorabilia at the same time. And you cannot get that much of that in this one [the mobile phone calendar]”* (female, age 75).

The participants noted that their home bound weekly/monthly calendars effectively met their needs, so they would not consider replacing them:

*“ I take out the weekly paper calendar every morning and I’m reading it, but you’re so custom to it that it’s just impossible to replace it”* (female, age 75).

Other participants described using between one and three calendars for different purposes:

*“I’m using a triple calendar, one at home on the wall, then this [showing a pocket paper calendar] and then that one [pointing at her mobile phone]”* (female, age 75).

Thus, a home bound weekly/monthly calendar was the point of departure for any other calendar that was added with participants supplementing their paper calendars with the new digital calendars.

The participants mainly used their mobile/smartphone at home as a telephone. At home, the mobile/smartphone was often placed in a special place, for example, in the hallway or the kitchen. Although some owned a smartphone, the smartphone was primarily used to make phone calls and not to access the Internet:

*“Actually, I have an older phone that I use most of the time. That is not used to browse the Internet. This old [shows the old phone], it’s a little easier to handle when you make phone calls”* (male, age 82).

Those that used pocket calendars and/or digital calendars through mobile/smartphones described these as easy to bring when being out and about, enabling the participants to reschedule activities and events in real time.

### Symbolic value

RemindMe, the interactive digital calendar with SMS reminders, seemed to hold different symbolic values. For some, RemindMe was a sign of modernity, symbolizing that the user was part of the rapid technology developing society. For others, RemindMe symbolized a loss of function and independence that comes with aging.

#### A sign of modernity

Participants perceived that using RemindMe was a sign of modernity. The fact they could learn to use RemindMe despite their age reinforced to them that they remained part of the larger technology society. The participants acknowledged that technology was important for their everyday life as technology was becoming a part of life for more and more people. Participants stressed there is a need to be open to digital solutions in everyday life to keep in touch with family and friends as well as for long-term planning to register events ahead of time such as family members’ birthdays or planned trips:

*“The advantage is that you can schedule far into the future and then* *it’s there. That is an advantage. I have already [spring] scheduled the autumn and that is an advantage. These things you can forget if you haven’t noted them”* (male, age 66).

Others emphasized that technology was a necessity in everyday life, so it may no longer be an option but rather a requirement to stay in touch with the fast pace of modern society, as expressed by one female participant:

*“Well it is coming [referring to technology] so you have to [learn], so it is best to try”* (female, age 79).

Reasons for using RemindMe reminders included feeling part of the twenty-first century and keeping in touch with society. For example, participants expressed that they were curious about new technical solutions and that it was exciting to try new technology, as illustrated by this conversation:

*“ I wanted to learn something new* (male, age 66) *…and find out if I could learn or not” (*female, age 66).

#### A sign of aging, dependence, and memory deficits

In contrast, there were also participants who perceived that RemindMe seemed to signal aging, dependence, and memory deficits. These participants emphasized talking about the usability and field of applicability for SMS reminders as well as responding to a reminder from older people with memory deficit, emphasizing that they were not one of these “older” people, yet:

*“I’m still rather clear in my brain. So maybe this isn’t something for me now. So, I thought that when you get older and maybe get a bit absent minded, in that case I think it could be good”* (male, age 76).

They suggested that RemindMe could be very useful for people with memory deficits or people who would need regular reminders for taking medication or performing other daily activities:

*“Yes, but as you said [referring to another participant’s statement], it’s a much better support for yes, disabled people as you said if you have had stroke. [.*. *.] You have familiar persons around who really need such a thing [RemindMe] and see the difference then. And then you can really think that there can be a big difference then”* (female, age 71).

These participants also speculated about future applications in senior care, stressing that these senior people might not have access to a computer or be experienced with computer or the mobile phone even though they did not have any problems with these issues themselves.

### Easy to learn but challenging to integrate in everyday life

Learning to use a digital calendar with the support of an introduction course and weekly phone support was described as easy. However, using RemindMe in everyday life was difficult for some participants, both in terms of integrating new habits into their daily lives and their abilities to handle the technology due to age-related difficulties. Use of RemindMe was described as embedded in daily routines and involved many habitual behaviours such as always having the mobile/smartphone with them and remembering to schedule activities, which influenced how the participants valued RemindMe.

#### Learning to use RemindMe despite age-related difficulties

Learning to use RemindMe was described by the participants as quite easy, especially for participants experienced with computers and mobile/smartphones. All the participants appreciated the introduction course and the participants stressed that the course enabled them to try different features of RemindMe at their own pace with the support of the research assistant. The participants particularly appreciated the research assistant’s weekly phone calls as these calls enabled them to ask questions and receive individual support based on their needs. Participants stressed that just knowing that the research assistant would call made them feel calm, and this was described as reassuring and was greatly valued. Age-related difficulties such as reduced hearing, vision, and fine motor skills were described as interfering with learning to use RemindMe. For example, participants with impaired vision stressed that small displays on their mobile phones made it difficult to read the reminders sent by RemindMe. In addition to difficulties reading SMS, several participants also mentioned that their decreased fine motor skills made it difficult to acknowledge the reminder by answering the SMS. That is, they experienced their fingers as too “clumsy” for the tiny buttons on the mobile phone. Nonetheless, these participants stressed that the introduction course and the ten-minute weekly support calls were sufficient for learning to use RemindMe.

#### RemindMe challenges habits

Nevertheless, incorporating RemindMe into the routines of everyday life brought new challenges for the participants, questioning both their habits and routines:

*“Well, I do not know if there was anything that was directly difficult, but it’s always a challenge to do something that you aren’t used to”* (female, age 70)*.*

Using RemindMe resulted in the participants having to change their mobile/smartphone habits as well as developing new routines for mobile/smartphone use. The participants noted that they were unfamiliar using their mobile/smartphone for receiving prompts and reminders in everyday life. Their only encounter with SMS reminders came from, for example, the dentist or the PRO as a reminder of a scheduled appointment or event, but these reminders were scheduled and sent by somebody else. Furthermore, participants stressed that they were not in the habit of carrying their mobile/smartphone inside or outside of the home:

*“I can leave it somewhere and then, then it’s lying somewhere beeping out of reach”* (male, age 81)*.*

Findings revealed that the participants had to adjust their routines to include the mobile/smartphone in their everyday life. The development of new routines or adjustments to already established daily routines were described by participants as a prerequisite for using RemindMe. The participants mentioned developing new routines that required scheduling reminders earlier so they could receive them before leaving home:

*“I’ve done so recently that the reminder comes earlier so I can answer it while at home”* (female, age 79).

Others who usually had the habit of placing their mobile phone in their handbag now put their mobile phone in their pocket, so it was easier to hear the signal or feel the vibration of the phone. Participants who usually did not write SMS noted that using RemindMe required them to actively use SMS. Often, they used a pencil rather than their fingers to push buttons when answering text messages, a strategy that seemed to make it easier to use the SMS feature.

### Active reminders in everyday life

Receiving active reminders through SMS for planned activities/events and actively acknowledging the reminder were described by the participants as enabling them to perform chosen activities in everyday life and as providing them with a higher sense of independence. Furthermore, receiving an active reminder through SMS and actively confirming the reminder supported the participants form new routines (e.g., with respect to medication), enabling the participants to gain control of the activity as well as take over the responsibility for the activity from significant others.

#### It talks to me

Reminders sent to the participants’ mobile/smartphones at scheduled times were described as providing a clear call for action without the person having to take the initiative to pick up the calendar and look for activities, actions that enhanced independence and performance of activities in everyday life, as these two quotes illustrate:

*“In an ordinary calendar you can write as much as you like, but if you don’t look into it, then it’s meaningless. Here [in RemindMe] someone talks to me, tells me it is time for something. So, this is great”* (male, age 66).

“I have a lousy memory. I write something down and then it is written, and I don’t memorize it here [point at the head] because it is stored there [in the calendar] and I have to see it [to remember]. But then, if I get a reminder of it *click sound* Yes!” (female, age 70).

For all participants, a new feature with RemindMe was to actively acknowledge the reminder by responding to a SMS. This feature was appreciated the most by participants with self-reported difficulties with memory. The participants described that actively responding to reminders through SMS increased the possibility of activities being performed more independently in everyday life. For example, one participant with memory difficulties after a stroke found this feature particularly helpful:

*“Having to actually answer a reminder [by SMS]*. *Yes, that’s when I remember it”* (female, age 66).

This participant described not always remembering whether she had performed scheduled activities, such as taking her medicine. By checking the feedback system in RemindMe, she was reassured that she had answered “Yes” to the received reminder, which was described as making her feel secure and in control over her daily medication intake.

Another participant described why answering the SMS was seen as positive:

*“You have to answer RemindMe. Other [reminders] just pop up, so to speak and you can click it away. The thing about this [RemindMe] is that you have to go in and write an answer”* (female, age 70).

The interactive part of RemindMe could also be experienced as support for relatives, especially spouses. One of the couples described that the active confirmation of reminders increased the likelihood of their partner independently performing activities, thereby gaining control of the activity, and as such taking over the responsibility for the activity from significant others. This resulted in spouses taking a step back and seemed to be able to relax their responsibilities of constantly reminding their spouse to perform an activity.

#### Not worth it for me

There were participants who had a difficult time recognizing the added value of the specific components incorporated in RemindMe. These participants described a limited need of reminders in their everyday life. They were rather dismissive than being opened to using RemindMe in everyday life. The main reason stressed for not using RemindMe is that there was no need for it or no interest in it. One participant stressed that RemindMe did not add any extra benefit for her:

*“First, you write it up in the paper calendar and then go and look at it all the time, then you have to type it in the computer and then answer the text message. I think it’s more work than it’s good for me”* (female, age 73).

In her case, the weekly/monthly calendar was sufficient as the digital calendar meant extra work without proving any clear benefit. Instead, RemindMe was perceived as extra stress receiving reminders through SMS when out and about, especially having to answer the SMS when in the middle of an activity. Other participants stressed that learning to use new technology was not worth it as the technology will probably be too hard to use when they actually need it:

*“As I see it, we are healthy seniors, but what about the day when we are not. How will we handle the technology then?”* (male, age 85).

Furthermore, these participants feared relying solely on technology in ways that removed the challenge of performing important cognitive skills.

## Discussion

Although it is well established that younger people often use different digital features in their tablet, computer, and mobile/smartphone [2], less is known about how senior people use digital features such as digital calendars. Moreover, little is known about how senior people perceive this technology and the barriers to learning and using a digital calendar. As suggested by Seeley et al. [9], this study focuses on how seniors use the technology in a real-world setting. Despite being experienced users of calendars and mobile phones in everyday life, most senior people in this study used more traditional weekly calendars, pocket calendars, and notebooks, what McDonald refers to as passive reminders [10]. Furthermore, in line with a recent study [32], the results demonstrate that owning a mobile/smartphone does not necessarily indicate that the full digital features are used in everyday life. Nimrod [33] explains this phenomenon using a pyramid of senior people’s incorporation of mobile phone and its different functions. At the bottom of the pyramid is making voice calls, followed by using basic functions such as SMS, and thereafter using Internet-based functions and media players. In terms of mobile/smartphone use, most participants in this study used only the basic features of a smartphone, such as making a phone call, preferably from home, and not necessarily all the new digital features included in the mobile/smartphone, including digital calendars, SMS, and the Internet.

The results of this study provide some insight into how senior people use new technology, information that could be used in clinical practice. Participants in this study described age-related difficulties such as reduced vision, hearing, and fine motor skills that influenced the use of digital features of the mobile/smartphone, in this case RemindMe. These results indicate that professionals need to account for physical and cognitive challenges when designing technological interventions for senior people [2, 16]. Some environmental aspects of mobile/smartphone use were also noted as influencing mobile/smartphone use, including adjusting sound levels in different environments. This usage indicates that professionals may need to support senior people to specify the contextual circumstances that influence their technology use and to develop strategies for overcoming barriers [34]. The results also demonstrate that people attached a symbolic value to the technology; some saw the technology as a symbol of being up-to-date and a part of the modern society and others saw using RemindMe as a mark of aging, dependence, and memory deficits. These issues point to the need for professionals to investigate the relationship between the technology used and the symbolic value attached to the technology by the user, as these attitudes may influence the use, expectations, and needs of the technology users. In addition, this study found that people’s habits play a crucial role when new technology is implemented. Therefore, healthcare providers working with senior people with cognitive impairments need to motivate and support the use of technology by discussing and developing strategies for how the new technology fits in with or can be incorporated into their daily routines.

Most people use a calendar to support memory of different activities/events to structure and organize everyday life [35, 36]. What calendar to use seems to be closely linked to personal habits. Habits are often deeply rooted and give structure and stability to people’s everyday life [37] and as a result might be difficult to change. This means that changing the type of calendar from passive to active often requires a need to change habits as well. Previous research has shown that habits are easier to develop or change when new behaviours or undertakings are connected to or complement an existing habit [34]. Assimilation is a process when new knowledge, such as using RemindMe, is added to established knowledge [38] such as using computers and mobile/smartphones. Thus, the fact that all the participants had some initial knowledge about computers and mobile/smartphones should be considered when interpreting the results.

Earlier studies have suggested interventions can be designed to change existing habits and to develop new habits. These interventions are described as action plans specifying where, when, and how one will engage in their intended behaviour and/or activity [34]. For this, users need to be motivated, for example, to receive and respond to an alert when experiencing reduced memory. A support person, invited by the user, can assist with instructions or can help with the scheduling for receiving prompts/reminders. This strategy can be used to introduce senior people to digital technology to overcome barriers such as the fear of using technology [39]. A promising finding in this study is that participants stressed that the short introduction course, the written manual, and the weekly phone support (based on the user’s needs and questions) were sufficient for delivering instructions and tips on using the technology. This support was also perceived as important emotional support, which has earlier been highlighted as important for learning and using technology [40].

A novel feature of RemindMe is the active confirmation of the reminder by responding to the SMS. The complementary action of actively acknowledging the reminder by answering a SMS from RemindMe may be a significant advantage for individuals with reduced memory because there is an action linked to the intended activity as well as the possibility to register whether the intended activity was performed. This advantage was reported by senior people with self-reported memory deficits. Thus, RemindMe’s feedback system appears to provide users with the possibility to self-monitor their registered activities/events, which in turn was described as providing evidence that the activity/event was done. This preliminary finding confirms the need to develop technology that provides flexible and easy ways for users to gain access to their data [7, 9]. Taken together, this indicates that RemindMe’s feedback system might enable users to self-monitor and enable professionals to provide individual support for performance of everyday activities using registered information [21]. However, replication of the present study’s findings with a larger sample of individuals with cognitive impairments is required before the effectiveness of RemindMe’s feedback system can be established.

### Limitations

The results of this study should be interpreted with the following limitations in mind. The participants were all recruited from different Swedish National Pensioners’ Organization (PRO). PRO provides a meeting place for seniors where they can meet new people and take part in a variety of cultural, social, and fitness activities. It is possible that these active senior people represent people who already have experience with technology such as computers and/or mobile/smartphones and thus limited difficulties using new technology as well as specific interest in taking part in developing new technology. Therefore, the findings may not be generalizable to senior people who might experience age-related and cognitive-related difficulties using technology or those in need of support to remember performing everyday activities. However, the present study provides initial understanding of how senior people, without cognitive impairments use RemindMe in everyday life. Future studies will evaluate how end users, senior people with cognitive impairment, use RemindMe.

## Conclusion

The findings reveal that senior people find RemindMe easy to learn although challenging to integrate in everyday life. Both age-related difficulties such as reduced sight, hearing, and memory as well as already established habits concerning mobile/smartphone and calendar use were cited as making use of RemindMe challenging in everyday life. Senior people described using mostly weekly/monthly paper calendars or pocket calendars at home or when out of the home, strategies that most often were sufficient for their needs. For senior people to make the effort to develop new routines for mobile/smartphone use, a prerequisite for using a digital calendar, they must be motivated to use digital calendars and perceive that active reminders will improve their everyday life. This was especially true for those people who felt active reminders were connected to disability and cognitive and physical deficits related to aging.

## Abbreviations

PRO: The Swedish National Pensioners’ Organization
RemindMe: An interactive digital calendar with mobile phone reminders
SMS: Short text messages
